# Identification of Key Processes Underlying Cancer Phenotypes Using Biologic Pathway Analysis

**DOI:** 10.1371/journal.pone.0000425

**Published:** 2007-05-09

**Authors:** Sol Efroni, Carl F. Schaefer, Kenneth H. Buetow

**Affiliations:** 1 National Cancer Institute Center for Bioinformatics, Rockville, Maryland, United States of America; 2 Laboratory of Population Genetics, National Cancer Institute, Bethesda, Maryland, United States of America; University of Sheffield, United Kingdom

## Abstract

Cancer is recognized to be a family of gene-based diseases whose causes are to be found in disruptions of basic biologic processes. An increasingly deep catalogue of canonical networks details the specific molecular interaction of genes and their products. However, mapping of disease phenotypes to alterations of these networks of interactions is accomplished indirectly and non-systematically. Here we objectively identify pathways associated with malignancy, staging, and outcome in cancer through application of an analytic approach that systematically evaluates differences in the activity and consistency of interactions within canonical biologic processes. Using large collections of publicly accessible genome-wide gene expression, we identify small, common sets of pathways – Trka Receptor, Apoptosis response to DNA Damage, Ceramide, Telomerase, CD40L and Calcineurin – whose differences robustly distinguish diverse tumor types from corresponding normal samples, predict tumor grade, and distinguish phenotypes such as estrogen receptor status and p53 mutation state. Pathways identified through this analysis perform as well or better than phenotypes used in the original studies in predicting cancer outcome. This approach provides a means to use genome-wide characterizations to map key biological processes to important clinical features in disease.

## Introduction

Biologic phenomena emerge as consequence of the action of genes and their products in pathways. Diseases arise through alteration of these complex networks [1]–[5]. In order to make mechanistic assertions that supplement current approaches to genome-wide analysis [6]–[9], we map canonical biologic pathways to cancer phenotypes. A total of 2011 Affymetrix GeneChip array hybridizations obtained from 9 different publicly accessible data sources [10]–[17] were analyzed. The hybridizations represented 70 different tumor types (1348 samples). Additionally 83 different types of samples of normal histology were included (663 samples). Expression levels were adjusted using RMA[18]. The definition of normal used here excludes uninvolved and/or tumor adjacent samples obtained from individuals with cancer.

The use of pathways as a framework for analysis is not in itself novel. These include the projection of known cancer genes and gene expression data onto pathways [19], [20]. What distinguishes the work presented here is the systematic evaluation of the interaction structure across predefined canonical networks. In measuring the state of the interaction it combines information from gene state and network structure. Multiple gene states may result in a common pathway score. Conversely, pathway scores may show greater differences than gene signatures.

### Approaches to Pathway Analysis

This investigation complements other work utilizing pathway information.

More specifically, Segal et. al. [6] defined biological modules and refined them to a set of statistically significant modules. They were able to use these modules to gain a better perspective on the different biological processes that are activated and de-activated in various clinical conditions. We note two main differences between what we present here and the work in Segal et. al. [6]: first, the biological modules used in the paper, although highly informative and useful, are internally defined within the paper. The determination of genes in these modules was derived from the same data to which they are later applied. The canonical pathways we use are externally defined independent from the data we analyze, represent current understanding in the field, and were not derived ad-hoc. Second, Segal et. al. do not make explicit use of the interconnections, or the network structure, that exists between genes that comprise biological modules. The scores for activity and consistency we present here depend on network structure and specific relations (such as inhibition and promotion) that are features of the network information.

Another important approach is that of Rhodes et. al. [21], in which the human interactome network is used to identify subnetworks activated in cancer. The approach Rhodes el. al. use, in contrast to the one presented here, does not attempt to computationally and algorithmically highlight differences in phenotypes by building a classifier around measurable network features. Instead, it generates subnetworks by their association with sets of genes identified through the over (or under) expression in each biological phenotype. Rhodes et. al. approach does make use of the network structure to build the subnetwork, but does not make further use in observing the co-expression or co-silencing of sets of genes, as is the case in the work presented here.

Bild et. al. [14] and Glinski et. al. [22] demonstrate that gene signatures determined by small set of pre-selected canonical pathways can distinguish tumor characteristics. In their work, they start with a limited set of pathways, (e.g. Bild et. al. use 5 pathways) and show that they differ in different phenotypes. As this approach starts with a small set of pathways the authors chose to examine, it does not have the capacity to discover new pathway associations with phenotypes. Unlike the current work, it does not employ an objective method to identify set of pathways that can discriminate phenotypes.

Gene set Enrichment Analyses [23] allows the authors to choose a set of genes and to determine their relative statistical significance in a list of genes that separate phenotypes. Gene set enrichment starts with the premise of individual genes as classifiers. Pathway membership is measured to assess combined contributions. Again, the method does not make use of the structure of the network, nor does it provide a systemic account for the combined knowledge of pathways to reduce to an optimal set of classifying processes. Since the method starts with the discrimination of single genes, it can only build on this statistical inference, and does not account for any differences that come from the inter-dependency of multiple gene interactions. For example, if gene A seems to permutate randomly in the two phenotypes and gene B seems to permutate randomly in the two phenotypes then each of the genes will score poorly in a statistical significance test. However, the score defined by their combined dependence (e.g. (if A then B)) might provide much greater discrimination.

The method by Tomfohr, et. al. [24] is perhaps the closest to the one presented here in that it looks at combined groups of genes and ranks them accordingly. However, Tomfohr, et. al. do not use the network structure knowledge to obtain scores, but instead perform Singular Value Decomposition (SVD) to choose a specific metagene, and define a pathway activity as the expression of that gene. As such, the result does not utilize the interdependence of the network as does the work presented above.

## Methods

### Evaluating a gene status:

Gene status in evaluating the network interaction is calculated from the observed data as one of two alternative states: down and up. To be able to identify whether a gene is in a “down” state or an “up” state, we look at its (RMA adjusted [18]) expression value in a sample, compared to the expression values of the same gene in all other samples. To be able to accommodate a multitude of probability distributions, we use a gamma distribution as the template to both the “down” distribution form as well as the “up” distribution, and redefine the problem as a mixture of two gamma distribution. The suppressed form often follows an exponential distribution, which is one particular case of a gamma distribution. The promoted state often follows a form similar to a normal distribution, which may be approximated by a gamma distribution of a large mean. Per every probe set measured by the microarray, we look at the expression distribution and try to fit this distribution into a mixture of two gamma distributions. We do this by using an Expectation-Maximization (EM) algorithm, iterating over the data in a manner that guarantees the increase of likelihood of fitting the data by the modeled distributions. In the case of two gamma distributions, we first divide the data into two groups: “down” values and “up” values. The number of genes in the “up” group is *N_U_* and the number of genes in the suppressed group is *N_D_*. The prior probabilities are therefore:
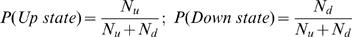



We assume each group distributes according to a gamma distribution:
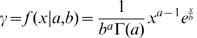



The objective of the EM algorithm is to provide us with maximum-likelihood estimates to the *a_U_, b_U_* values for the promoted group and to the *a_D_, b_D_* values for the suppressed group. Additionally, it computes the maximum-likelihood estimates of the mixture coefficients, η_1_, η_2_.

We assume that the expression distribution of each gene is either coming from a mixture of two distributions (one for the “up” case and one for the “down” case) or from a single distribution (for example, when the gene is “up” in all the samples we have). We determine the number of underlying distributions (one or two) using the EM algorithm in combination with a model selection method, see below.

To find the maximum of the log likelihood, we need to find the maximum of the auxiliary function *Q* [25]:
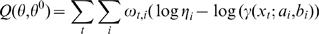
where
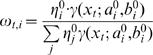
Here, *θ* is the collection of parameters that define the distribution, and the superscript 0 designates magnitudes that had been determined in the previous iteration.

To find maxima, we differentiate *Q* with respect to the model parameters, and compare to zero.
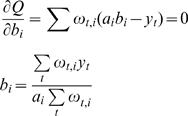
And the coefficients
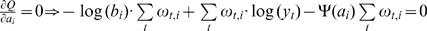
where Ψ(*x*) is the psi function 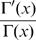.

Using a Lagrange multiplier to incorporate the constrain 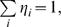
we have to maximize the target function
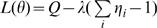
with respect to the *η_i_*, we derive
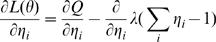
and obtain
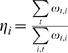
We solve this numerically (using Matlab®) in every iterative step, until we reach some predefined convergence criterion.

### Choosing an optimal number of distributions:

Obviously, the more distributions we take as our basis for the overall distributions, the better fit we have for the data and the better the likelihood will be. Consider, for example, as many distributions as there are data points. That would fit the data exactly and produce maximal likelihood. To overcome this, and to be able to choose an optimal number, we compare models with different number of distributions using the Bayesian Information Criterion (BIC)[26], computed as
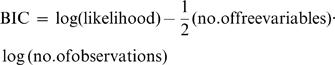
This cost function compensates for the additional increase in complexity. The statistical model chosen is the one with the largest BIC.

**Figure 1:**
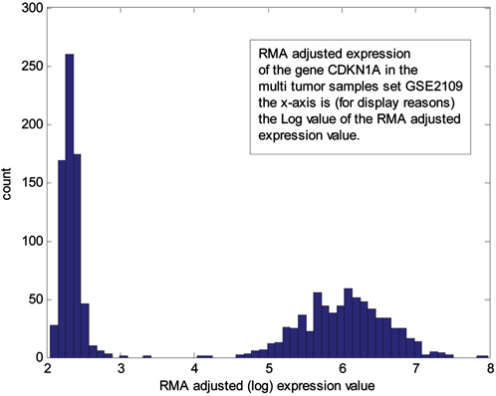
An example to the distribution of gene expression and its resemblance to a mixture of two Gamma distributions. The Up/Down calls for gene states are based on an expression value classified as residing in one of the two distinct distributions.

**Calculate** 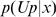


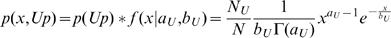
And similarly:
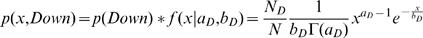
But we need the probability of being in the “promoted” state for a specific expression value:
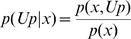
And since
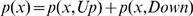
we can obtain the needed values by:
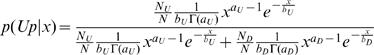
For example, the expression of the gene CDKN1A in the dataset [13] (a collection of 698 tumor samples) follows this distribution (see Figure 1):

The two distinct distributions (Down and Up) are evident and the algorithm gives the parameters for the two gamma distributions.

### Pathway activity and pathway consistency

**Figure 2:**
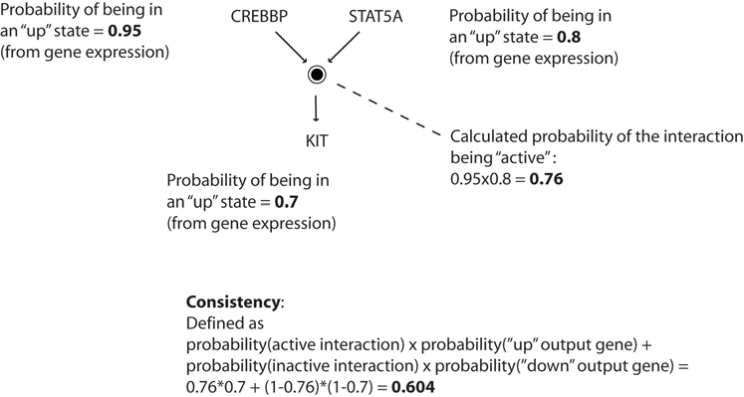
An example to calculating the consistency and activity of a single interaction. See Methods for details.

1. Pathway consistency score: To determine the pathway consistency score of a given signaling pathway in a sample, we follow these steps:
    - Every pathway is a collection of interactions. Input genes and output genes define each interaction. For each interaction in the pathway, we first look at the input genes and determine, for each such gene, the probability of being in a “down” or “up” state (see “gene state” above)
    - We then determine the probability of the materialization of the specific interaction as the joint probability of all needed components (genes)
    - Next, we look at the molecular output of the interaction. Usually, this output is a list of genes, for which we establish the probability of being in a “down” or “up” state (see “gene state” above)
    - Next, we calculate the likelihood of the output gene(s) being in one of the two states, under the given probability of the interaction (calculated in (b))
    - Lastly, to obtain the pathway consistency score, we calculate the consistency score for every interaction in the pathway and average the scores over all the interactions for which we were able to obtain a score. In Figure 2 we show an example to calculating the consistency value of an interaction taken from the pathway “Signaling events mediated by Stem cell factor receptor (c-Kit)”, one of the NCI-Nature Curated pathways from the Pathway Interaction Database (PID)[27]. The specific steps to calculate consistency in this example are:
        1. Establish probabilities to all genes involved in the interaction. This is done according to the steps described below (see “gene state” section). The values we obtain are: P(CREBBP) = 0.95; P(STAT5A) = 0.8; P(KIT) = 0.7
        2. Calculate the joint probability of an active interaction. Since the input molecules to the interaction are not co-dependent, the joint probability of the interaction is P(CREBBP)×P(STAT5A) = 0.95×0.8 = 0.76
        3. Calculate the likelihood that the output molecule is the result of the interaction. Since the molecule is solely dependent on the interaction the likelihood is straightforward:
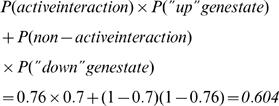

        4. Iterate this computation throughout all interactions in the pathway. The final score of a pathway is an average over all interactions.
2. A pathway activity score is the average over activity of interactions in a pathway. For example, in the previous example, the interaction activity is 0.76. The main advantage to calculating pathway activities on top of pathway consistencies is that activities can be calculated even when there is not enough data to work with the output, as is the case, for example, when the interaction is based on activating or modifying molecules without the generation of a novel molecule as output. In such cases, we can still calculate the activity, although consistency loses its meaning.

### Choosing a minimal set of pathways to classify phenotype

As we obtain pathway activity and consistency scores for each pathway, we are able to transform the representation of each bio-sample from a list of gene expression measurements into a novel representation, displaying each sample with the collection of pathway activity and consistency scores. As we use this representation to distinguish between phenotypes, we wish to find the minimal set of pathways scores that is able to make the distinction between phenotypic classes. We use feature selection to choose an optimal minimal set (see Results). We used different methods of feature extraction and feature classification [28], [29], including forward selection, backward selection, and floating search [29]. These methods help in eliminating pathway scores that do not contribute to making the distinction and highlighting specific pathways that together achieve an optimal classification rate.

### Pathway metric to predict outcome

Representing each bio-sample using its pathway metrics allows us to look for patterns in the collection of pathways. By using clustering algorithms, we see that pathway metric values segregate samples into groups. If we look at the survival patterns of these groups, we see that in some cases and for some pathways, the groups correlate with distinct patterns of survival.

## Results

The analysis applied here treats a pathway as a network of genes whose interactions are logically evaluated in the pathway context to generate sets of scores. Biologic pathway structure information was obtained from public sources [27], [30], [31].

Each pathway is assessed for consistency and activity. A pathway consistency score is calculated as the average likelihood of the logical consistency of the collection of interactions given the calculated states of the genes (see Methods). A pathway activity score is calculated as the average likelihood of the pathway's individual interactions being active given the calculated gene states. Using basic principles of machine supervised learning [28], [29] a classification algorithm that distinguished each oncogenic phenotype (e.g. cancer sample verse normal) was generated and validated. Based on simplicity and comparability of alternative approaches tested, a Bayesian linear discriminant classifier was used.

**Figure 3:**
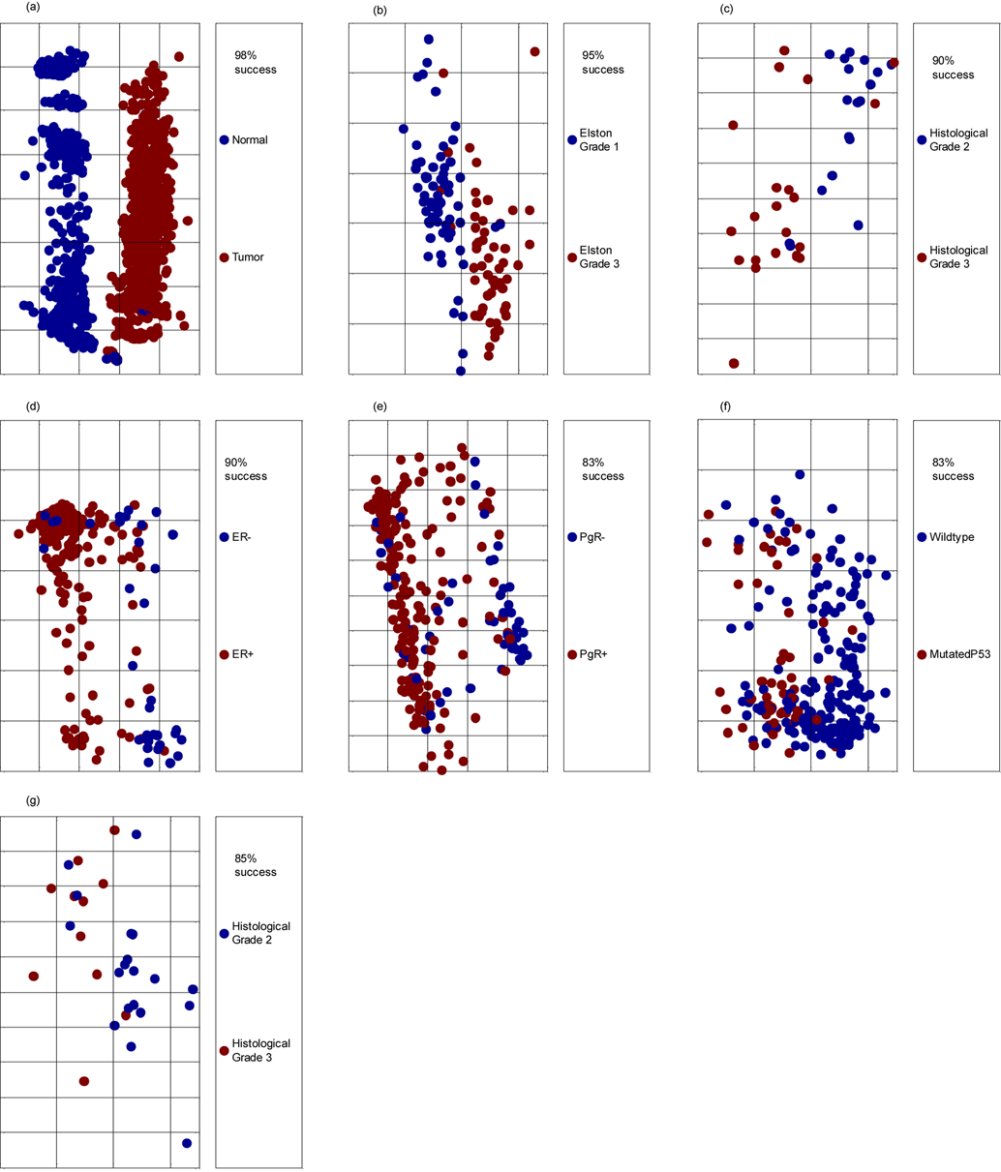
Classification results of the different classifiers tried. Each panel in the figure corresponds to a different phenotypic difference, according to panel captions. The horizontal axis in each panel corresponds to the one-dimensional projection calculated by the classification algorithm, that signifies distance between biological samples, according to the multi dimensional pathway metrics. The vertical axis is a jitter scatter of the samples to enable a clear view of the separation.

First, a classification algorithm was derived to distinguish diverse cancer phenotypes from normal phenotype tissues. A classifier derived from an 1800 sample training set (10-fold validation) demonstrated 98% success in an independent validation test set of 211 samples (see Figure 3 and Table 1).

**Table 1:** Pathway names and classes.

Classification	Pathway name and metric (A-activity, C-consistency)	Pathway Title
Normal/Tumour separation	Trka Receptor (A)	Trka Receptor Signaling Pathway
	DNA Damage Apoptosis (A)	Apoptotic Signaling in Response to DNA Damage
	Ceramide (A)	Ceramide Signaling Pathway
	Telomerase (A)	Overview of telomerase RNA component gene hTerc Transcriptional Regulation
	CD40L (A)	CD40L Signaling Pathway
	Calcineurin (A)	Effects of Calcineurin in Keratinocyte Differentiation
Separation of Histological Grades 1/3 in breast cancer tumour	NGF (A)	Nerve Growth Factor Pathway (NGF)
	Ras (A)	Ras Signaling Pathway
	Circadian Rhythms (A)	Circadian Rhythms
	IL-7 (A)	IL-7 Signal Transduction
Separation of Histological Grades 2/3 in breast cancer	Sonic Hedgehog (A)	Sonic Hedgehog Receptor Ptc1 Regulates Cell Cycle
	Csk Activation (A)	Activation of Csk by cAMP-dependent Protein Kinase Inhibits Signaling through the T Cell Receptor
	ChREBP Regulation (A)	ChREBP Regulation by Carbohydrates and cAMP
	Trka Receptor (A)	Trka Receptor Signaling Pathway
	HDAC and CaMK (A)	Control of skeletal myogenesis by HDAC and calcium/calmodulin-dependent kinase (CaMK)
Separation of ER+/ER− breast cancer samples	Th2 activation (A)	GATA3 participate in activating the Th2 cytokine genes expression
	Lipid Synthesis (A)	SREBP Control of Lipid Synthesis
	ER modulation (A)	Pelp1 Modulation of Estrogen Receptor Activity
	LIS1 dependent migration (A)	Lissencephaly gene (LIS1) in neuronal migration and development
	Erk1/Erk2 MAPK (A)	Erk1/Erk2 MAPK Signaling Pathway
Separation of PgR−/PgR+ breast cancer samples	Th2 activation (A)	GATA3 participate in activating the Th2 cytokine genes expression
	Bone remodeling (C)	Bone remodeling
	Mucosal Healing (A)	Trefoil Factors Initiate Mucosal Healing
Separation of P53 mutated/P53 wildtype breast cancer samples	Cdc25 and chk1 (A)	cdc25 and chk1 Regulatory Pathway in Response to DNA damage
	Neuronal Survival (A)	Role of Erk5 in Neuronal Survival Pathway
	Postsynaptic Differentiation (C)	Agrin in Postsynaptic Differentiation
	Regulation of Splicing (A)	Regulation of Splicing through Sam68
	T cell activation (A)	The Co-Stimulatory Signal During T-cell Activation
	ACH Receptor Apoptosis (C)	Role of nicotinic acetylcholine receptors in the regulation of apoptosis
Separation of histological grades 2/3 in colon cancer	Telomerase (A)	Overview of telomerase RNA component gene hTerc Transcriptional Regulation
	NFkB activation (A)	NFkB Activation by Nontypeable Hemophilus Influenzae

Since linear classifiers turn each of the pathways in the problem into a variable in the classifier, it is possible through feature analysis to identify subsets of classifier variables (pathways) that, as a group, distinguish the phenotypes with high accuracy. Feature selection was used to identify a set demonstrating the optimal 98% accuracy of the original classification in the validation sample analysis. It is composed of the activity scores of six pathways: Trka Pathway, DNA Damage pathway, Ceramide Pathway, Telomerase Pathway, CD40L Pathway and Calcineurin Pathway.

Cancer is a disease of great phenotypic and molecular heterogeneity. Even within a given organ site, phenotypic heterogeneity is associated with significant differences in cancer outcome. It is therefore of additional interest to identify molecular processes that underlie the phenotypic differences and that predict outcome. We therefore derived signatures for a variety of subtypes of breast cancer. These subtypes include: histologic grade (Elston grades 1 vs. 3, or grades 2 vs. 3); P53 status (mutated/wild type); estrogen receptor positive/negative status (ER+/−); and progesterone receptor positive/negative status (PgR+/−). The performance of the classifiers is displayed in Figure 3. In all cases, classifiers with a small number of pathways (three to six) achieved a high level of accuracy (83% to 95%). Table 1 shows the different pathway groups that classify different phenotypes.

**Figure 4:**
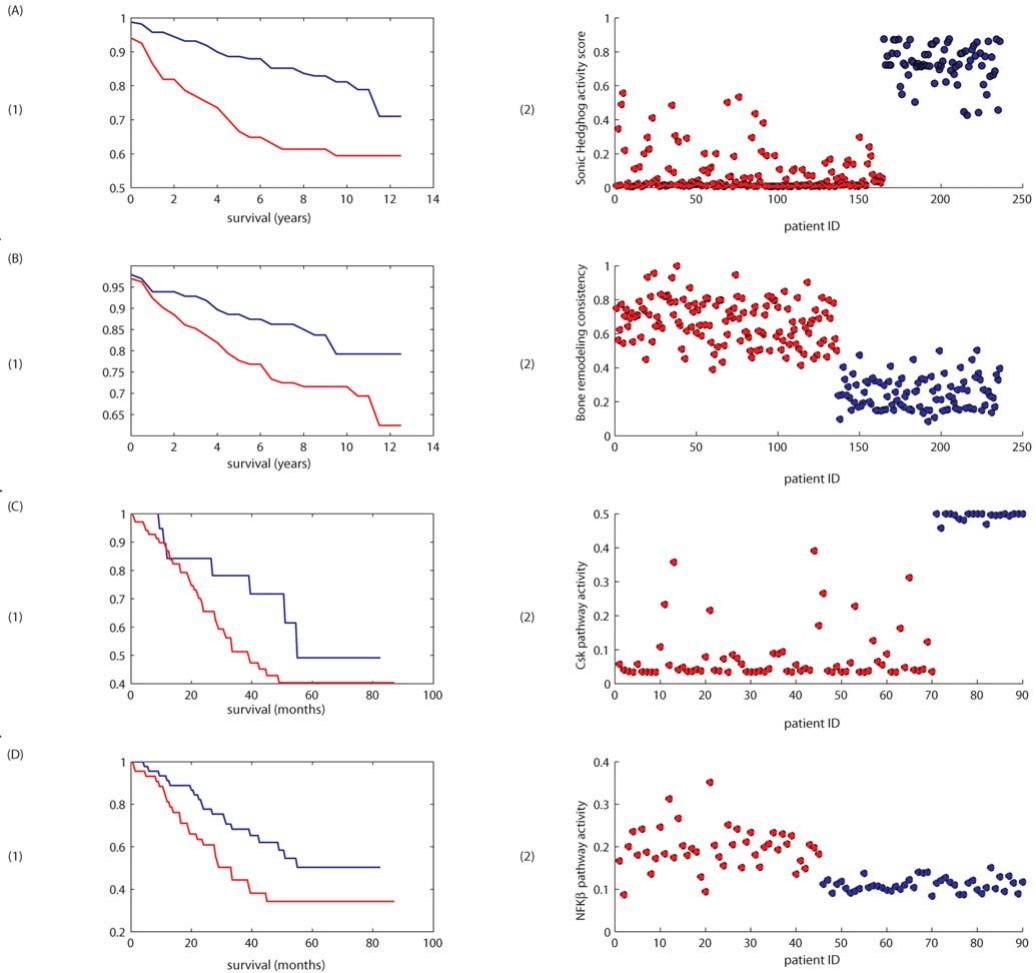
Examples of the stratification of survival plots and their immediate connections to pathway activity/consistency. (A) (1) Kaplan-Meier survival plot of breast cancer patients from [15], stratified according to clustering based on pathway activity. Panel (2) in (A) shows the activity score of the Sonic hedgehog pathway colored according to affiliation with either of the accordingly colored survival curves in (1); (B) The same analyses done with breast cancer patients from [15], based on the pathway Bone Remodeling (see text for pathway choice). (C) Kaplan Meier survival plots of lung cancer patient data from [17], stratified according to activity of the Csk pathway and the (D) NFKβ pathway. In every panel, the (2) sub-panel shows the most influential pathway metric out of the group of stratifying pathways. This does not mean that the pathway represented is responsible for the entire separation into two groups.

We next evaluated the ability of the cancer subtype-specific signatures to stratify the 236 breast cancer samples by outcome. Following unsupervised clustering of the cancer samples using the pathways identified above, Kaplan Meier analyses was performed (Figure 4). In three cases, a single pathway from the sub-type signature significantly predicted outcome: the Circadian Rhythms pathway, from the grade 1/3 signature (P = 2.9E-11); the Sonic Hedgehog pathway, from the grade 2/3 signature (P = 4E-8); and Agrin in Postsynaptic Differentiation, from the P53 signature (P = 4.6E-7). The three pathways in the PgR+/− signature separated the samples into two groups with a P value .0001, with the Bone Remodelling pathway accounting for most of the effect. In addition, the five pathways in the ER+/− signature separated the samples into two groups with a P value of .004, with the SREBP pathway accounting for most of the effect.

It is important to note that a number of findings in the literature emerge independently from our pathway analysis of the breast cancer samples. As the importance of the ER+/− distinction in management of breast cancer is well established, we looked at each of these subgroups separately. It has been observed [32] that the Trka Pathway (identified in both the generic oncogenic signature and the grade 2/3 signature) plays a significant role in ER- cases. Our analysis shows that the generic oncogenic signature separates the ER- samples into two groups (P = 4.6E-9) with the Trka pathway accounting for most of the effect, high activity of this pathway correlating with poor prognosis. Likewise, it has been observed [33], [34] that beta-catenin plays a significant role in the response to tamoxifen, a standard treatment for ER+ disease. To analyze the nature of the tamoxifen-induced response, we derived a classifier to distinguish the ER+ cases that had been treated with tamoxifen from those cases that had not been so treated and then used the pathways in the resulting signature to cluster the cases by outcome. The Beta-catenin pathway emerged as the most significant (P = 1E-13) pathway in predicting outcome.

It has long been suggested that molecular classifications of cancer may have the capacity to transcend organ or tissue-specific definitions. More specifically, it has been suggested that molecular definitions that reflect the universal properties of cell type or ontology and that underpin a common molecular etiology may emerge across organ site definitions. To assess whether the signatures observed above in cancer of breast epithelium may generalize to other cancers, we examined their capacity to predict phenotypes in lung and colon cancer. We applied signatures derived from the breast cancer subtypes to cluster the lung cancer outcomes (Figure 4). Pathways predicting outcome included the IL-7 Pathway (P = .002) and the Csk Pathway (P = 3E-11). It has been previously noted that these pathways have been linked with outcome in lung cancer [35], [36]

Lastly, we examined the general oncogenic signature's capacity to predict organ-site specific outcome. Interestingly, the signature pathways separated the 236 breast cancer samples into five different survival subgroups (P = 2E-8) and the 90 lung cancer samples into two different subgroups (P = 5E-17).

## Discussion

The above results suggest that using the pathway as the unit of analysis can augment current individual gene based approaches to mapping phenotype to underlying molecular process. Objective identification of processes previously associated with phenotypes utilizing genome-wide datasets provides partial validation of the observed results. Newly observed process mappings to phenotypes, however, clearly require either verification from independent data sets or experimental confirmation.

The observations made through this analysis are provocative. Many of these pathways (e.g. apoptosis, telomere maintenance) have been previously described as universal components of oncogenesis[2]. Additionally, processes are identified that may underlie common cancer related phenotypes, such as inflammation. Interestingly, novel pathways are also identified as part of the general oncogenic signature as depicted in the six pathways collective (e.g. Ceramide and Calcineurin pathways). Recent interest in Ceramide supports this hypothesis. Ceramide has been long known to be involved in apoptosis [37]–[39] and recent work is looking at the relevancy of ceramide in cancer [40]–[42] and in cancer therapy [43], [44]. Similar interest has been developing in calcineurin. Whereas interest was previously confined to its activity in immune response, it is now becoming recognized as a predominant participant in oncogenesis [45], [46]. The combination of this set of pathways may define key processes that are characteristic of a universal progenitor cell type.

Conversely, the pathway analysis of cancer sub-phenotypes may also provide novel mechanistic insights that reveal underlying biology. For example, tamoxifen is effective in treating some cases of ER+ breast cancer. In these cases, tamoxifen must be affecting the activity of interaction networks. It is therefore logical to hypothesize that there will be observable differences in network activity between those cases where tamoxifen is effective and those cases where the drug is not effective. Our approach uses pathway signatures to predict variance in outcome, which is taken as the measure of drug effectiveness. We speculate that our approach can reveal those networks that are both differentially activated in response to treatment with tamoxifen and important to tumor growth and sustainability.

The approach applied here has parallels to the use of gene maps for translating phenotypes into the molecular domain. First, pathway models represent a reproducible framework that can be tested across studies and extended as further knowledge becomes available. Also, the pathways and their structure provide a higher order construct for assessing the role of genes.

Each interaction within a pathway requires the contribution of multiple gene observations. Each single gene activity level contributes only in the context of other genes participating in an interaction within the process network. This is demonstrated by the observation that we were unable to derive effective classifiers, directly from the gene-state values alone (for the genes composing the main six pathways).

**Figure 5:**
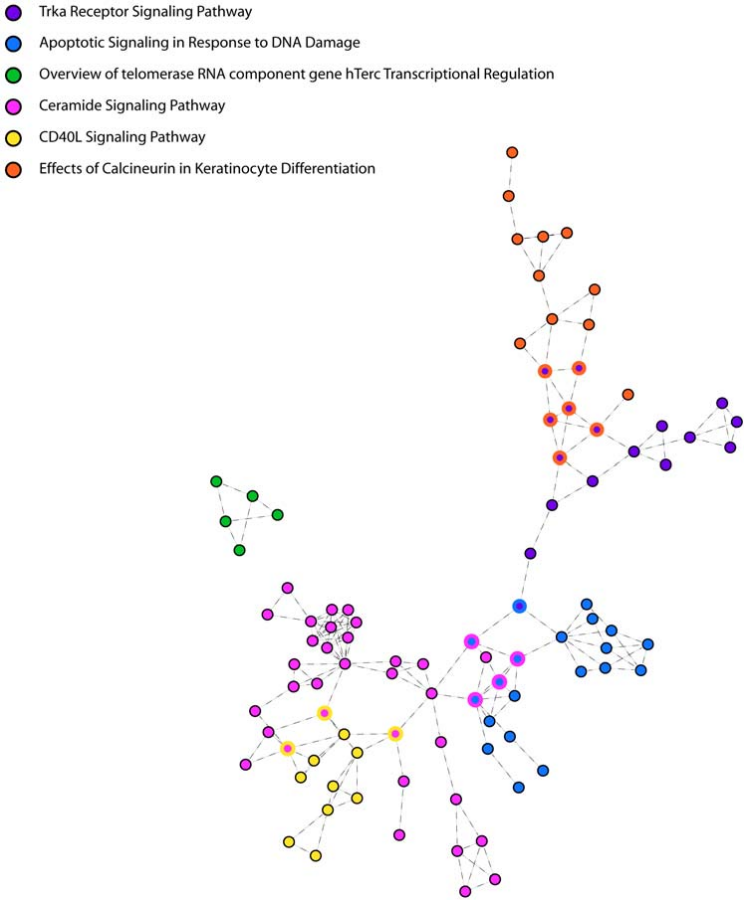
The sub network formed by the six pathways that together create the normal/tumor classifier. The joined pathways color shared nodes.

It is also interesting that five of the six pathways we use to classify normal and tumour samples form a single connected network (Figure 5, the telomerase pathway remains unconnected). This interconnection may provide novel opportunities for developing interventions. Knowledge of the connections may suggest alternative targets that would have multiple pathway effects. Minimally, it may permit the identification of complexities associated with target selection prior to intervention design.

It is understood that the probabilistic classification of genes into alternative states of down and up is a simplification of much greater complexity patterns of gene behaviour and action. However, empiric evaluation of the observed data finds that gene expression patterns commonly can fit one of two alternative expression level distributions. Moreover, such simplification has proven valuable in other research domains. For example the simplification that abstracts digital logic from the underlying continuous flow of electrons in integrated circuits has enabled the design of devices of staggeringly complex functionality [47].

It is clear that current knowledge of biologic pathways is incomplete and imperfect. As such, processes identified are almost assuredly not the only factors influencing the phenotypes of interest. Nevertheless, where processes are identified, they serve as important targets for further investigation. Moreover, the process-oriented approach allows one to distinguish which components of the complex networks in which genes participate are differentially contributing to a phenotype of interest. The combined use of activity and consistency score permits the discrimination of processes activated because of the phenotype versus those whose logic differs between phenotypes. The latter (consistency), potentially is causally attributable to the phenotype and suggests candidates that have been altered. However, utilizing gene expression data, consistency scores can only be calculated for interactions involving transcription events, limiting their discriminatory power.
